# Age- and BMI-related variations of fat distribution in sacral and lumbar bone marrow and their association with local muscle fat content

**DOI:** 10.1038/s41598-020-66649-8

**Published:** 2020-06-16

**Authors:** Egon Burian, Jan Syväri, Michael Dieckmeyer, Christina Holzapfel, Theresa Drabsch, Nico Sollmann, Jan S. Kirschke, Ernst J. Rummeny, Claus Zimmer, Hans Hauner, Dimitrios C. Karampinos, Thomas Baum, Daniela Junker

**Affiliations:** 1 Department of Diagnostic and Interventional Neuroradiology, Klinikum rechts der Isar, Technical University of Munich, Ismaninger Str. 22, 81675 Munich, Germany; 2 Department of Diagnostic and Interventional Radiology, Klinikum rechts der Isar, Technical University of Munich, Ismaninger Str. 22, 81675 Munich, Germany; 3 Department of Nutritional Medicine, Klinikum rechts der Isar, Technical University of Munich, Ismaninger Str. 22, 81675 Munich, Germany

**Keywords:** Anatomy, Biomarkers, Endocrinology, Medical research

## Abstract

This analysis investigated the age- and BMI-related variations of fat distribution in sacral and lumbar bone marrow and their association with local muscle fat content in order to detect fat distribution patterns and variations in healthy adults using proton density fat fraction (PDFF) measurements. A six-echo 3D spoiled gradient-echo sequence was used for chemical shift encoding-based water-fat separation at the sacral and lower lumbar region in 103 healthy volunteers. PDFF values of the sacrum, 5^th^ lumbar vertebral body, the gluteal and paraspinal muscles were determined. Correlation with age was significant (*p* < 0.05) for PDFF of the sacrum (men (m): *r* = 0.58; women (w): *r* = 0.54), L5 (m: *r* = 0.58; w: *r* = 0.54), the gluteal (m: *r* = 0.51; w: *r* = 0.44) and paraspinal (m: *r* = 0.36; w: *r* = 0.49) muscles in both genders. BMI correlated significantly with the paraspinal musculature in men (*r* = 0.46) and women (*r* = 0.33). Correlation testing revealed significant correlations (*p* < 0.05) between the two osseous (m: *r* = 0.63, w: *r* = 0.75) and the muscle compartments (m: *r* = 0.63, w: *r* = 0.33) in both genders. Bone marrow and muscle fat infiltration patterns were not significantly associated with each other at the sacral and lower lumbar spine region. The presented data suggest that the two compartments may have distinct pathophysiological fat infiltration patterns. However, further clinical studies are needed to support the results.

## Introduction

Compositional variations of bone marrow as well as intramuscular fat have been shown to be related to both age and body composition. It is known that in diseases like osteoporosis or sarcopenia fat content of bone marrow is elevated and the fatty infiltration of muscular tissue is increased^1,2^.

In senescence macroscopic changes in regard of bone marrow adiposity and muscular fatty infiltration as well as decreasing unsaturation and increasing saturation levels of fatty acids have been observed^1–3^. As osteoporotic fracture risk significantly increases due to aging, understanding the pathophysiology and subsequent structural alterations accompanying muscle loss and increased bone marrow adiposity is of high scientific but also of substantial clinical and economic value^4,5^. Besides aging, an unfavorable body composition has been shown to be associated with bone marrow composition, bone health and fracture risk^6,7^. Against the background of an aging population in context of the demographic shift and both obesity and undernutrition being growing health problems worldwide, exploring the connection of structural changes in muscular tissue and bone marrow and their interactions with each other will gain importance in the near future.

But what is the immanent clinical use of evaluating and comparing ectopic fat distribution patterns in the paraspinal and gluteal musculature and the fat content of the vertebral bone marrow in a healthy study collective besides elucidating the interplay of osseous and adjacent muscle tissue on a fundamental level? First, vertebral bone marrow fat fraction has previously been shown to increase fracture discrimination power when used with bone mineral density (BMD)^8^. As shown in previous studies, bone marrow PDFF values show specific distribution patterns inter- and also intra-individually, depending on the topography within the lumbar spine, with increasing PDFF from cranial to caudal (L1 to L5)^3,9–11^. Furthermore, an age-dependent correlation between the structural composition of the local lumbar musculature and vertebral bone marrow fat has been described recently^12^. A systematic analysis of the functional impact associated with these pathophysiological processes using isometric strength measurements showed that an increased fatty infiltration of the paraspinal muscles significantly correlated with reduced muscle strength^13,14^. Moreover, several recent studies elucidated the connection between a sedentary lifestyle, muscle volume loss and increasing intramuscular fat, decreasing BMD and associated evolving facture risk and delayed bone healing processes^15,16^. But not only fracture risk and muscle strength, the latter being connected to frailty in elderly persons, are associated with bone marrow composition and local muscle fat content. In previous studies ectopic, intramuscular fat accumulation has been shown to be associated with decreasing insulin sensitivity and metabolic diseases^17,18^. Furthermore, vertebral bone marrow fat content has also been shown to be correlated with insulin resistance in postmenopausal women diagnosed with Type 2 Diabetes, which suggests the use of vertebral PDFF for screening of obese subjects with a tendency towards developing metabolic syndrome^19^.

Structural composition of vertebral bone marrow and muscle can be assessed using magnetic resonance spectroscopy (MRS) and chemical shift encoding-based water–fat magnetic resonance imaging (MRI)^20,21^. This allows for calculating surrogate values like the proton density fat fraction (PDFF), and with MRS also the fat (un)saturation levels^9,20–23^.

Non-invasive muscle status assessment with chemical shift encoding-based water-fat MRI would allow for early interventions in subjects showing local fat accumulation levels. With obesity, osteoporosis, their associated diseases and ageing populations being on the rise, an accurate assessment tool identifying patients with abnormal values would be of benefit for clinicians in the future^24,25^. However, the age- and BMI-related variations of the sacral bone marrow composition and their association with local muscle fat content have not been investigated yet.

Thus, the purpose of this analysis was to investigate lower lumbar and sacral bone marrow fat content based on chemical shift encoding-based water-fat MRI in men and women and their correlation with the composition of local musculature and analyze compositional variations with regard to age and BMI.

## Materials and Methods

### Subjects

103 volunteers (72 women and 31 men) were recruited at the Institute for Nutritional Medicine, Klinikum rechts der Isar, Technical University of Munich, from a large observational study aiming at evaluating determinants of basal metabolic rate^26^. The study protocol and procedures were approved by the ethical committee of the Faculty of Medicine of the Technical University of Munich, Germany. Subjects were screened for eligibility and included if age was equal to or greater than 18 years, they had no history of severe diseases or surgery within the last three months, acute physical impairment or standard contraindications for MRI. All subjects gave written informed consent prior to study inclusion. All subjects underwent an MRI including a 3D spoiled gradient echo sequence at the upper pelvic/ gluteal region. On the basis of the acquired sequence the measurement of PDFF values was conducted.

### MR imaging

All subjects underwent MRI at 3T (Ingenia, Philips Healthcare, Best, The Netherlands). Subjects were positioned head-first in a supine position. An axial six-echo multi-echo 3D spoiled gradient-echo sequence was applied for chemical shift-encoding based water-fat separation using anterior and posterior coil arrays. The sequence acquired the six echoes in a single TR using non-flyback (bipolar) read-out gradients and covered the gluteal region with the following imaging parameters: TR/TE1/ΔTE = 8.2/1.24/1.0 ms, flip angle = 5°, FOV = 400 × 300 × 140 mm^3^, acquisition matrix size = 268 × 200, acquisition voxel size = 1.5 × 1.5 × 1.5 mm³, SENSE with reduction factor = 2.5 × 1.0, receiver bandwidth = 1413 Hz/pixel, 2 averages, scan time = 2 min 1 s^12,27,28^.

### Vertebral bone marrow and muscle fat quantification

**Figure 1:**
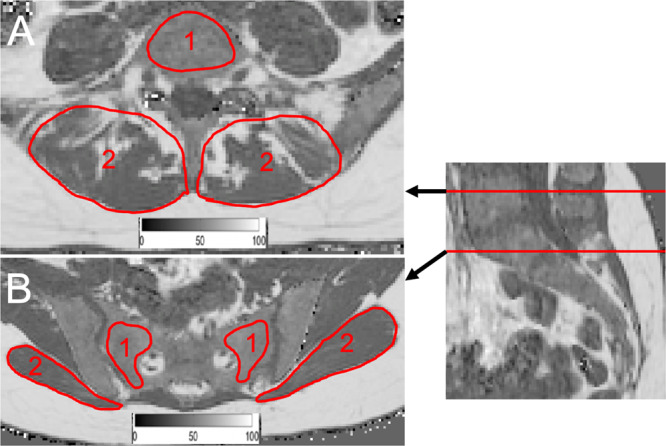
(**A**) PDFF map of a 27-year-old woman (BMI: 26.7 kg/m^2^): Representative segmentation of the fifth lumbar vertebral body (1) and the local paraspinal musculature (2). (**B**) PDFF map of a 27-year-old woman (BMI: 18.9 kg/m^2^): Representative segmentation of the sacrum (1) and the gluteal muscles (2). A color-coded scale bar displaying a spectrum from 0 to 100% can be seen at the bottom of images (**A**,**B**). PDFF: proton density fat fraction, BMI: body mass index.

The gradient echo imaging data were processed online using the fat quantification routine of the MR vendor (Philips Healthcare, Best, Netherlands). PDFF maps were generated using a complex-based water-fat separation algorithm that accounts for known confounding factors including a single T2* correction, phase error correction and the consideration of the spectral complexity of fat using the multi-peak fat spectrum model of Ren *et al*.^29^. Segmentations were performed by a radiologist using the free open-source software Medical Imaging Interaction Toolkit (MITK, developed by the Division of Medical and Biological Informatics, German Cancer Research Center, Heidelberg, Germany; www.mitk.org). The lateral masses of the sacrum, the fifth lumbar vertebral body (L5), the gluteus maximus muscles and the paraspinal muscles were included in the analysis and manually segmented bilaterally in the PDFF maps in 5 slices at the height of L5 and the first sacral vertebral body level, respectively. At the level of L5 the region of interest (ROI) was placed in the center of the vertebral body, thus avoiding the accidental inclusion of Modic endplate changes. PDFF values of each resulting ROI were recorded and the average value of both sides was calculated. Figure 1 exemplarily shows ROI depiction for the levels L5, S1, the paraspinal and gluteal muscles. Due to artifacts in the gluteal muscles, 7 cases had to be excluded from the gluteal muscle analysis, resulting in 96 evaluable cases (29 men and 67 women).

### BMI calculation

Height was measured without shoes in a standing position using a stadiometer (Seca, Hamburg, Germany), and reported to the nearest 0.5 cm. Weight was assessed on the day of the MRI scan. BMI was calculated as the quotient of weight in kg and height in meter squared (kg/m^2^).

### Reproducibility of PDFF measurements

Ten randomly selected subjects (3 males, 7 females) from the study population were used to determine the reproducibility of the PDFF measurements. All bone marrow and muscle segmentations as outlined above were performed a second time by the same radiologist 8 weeks after the initial segmentations. Intra-reader reproducibility error was expressed as root mean square absolute precision error in percent (absolute units) according to Gluer *et al*.^30^. The error amounted 0.77% for the sacrum, 0.64% for the gluteal muscles, 1.03% for the vertebral body L5 and 0.48% for the paraspinal muscles. The calculated results indicate a high reproducibility of the extracted PDFF measurements. Furthermore, the reproducibility of the described methods was shown in previous studies for the vertebral bone marrow as well as for the muscular compartments^13,22^.

### Statistical analysis

All statistical tests were performed using SPSS (Version 25, SPSS Inc., Chicago, IL, USA). All tests were performed using a two-sided 0.05 level of significance. Graphpad Prism Version 7 (Version 7.0, GraphPad Software Inc., La Jolla, CA, USA) was used for generating Fig. 3. Before performing further statistical analysis the Kolmogorov-Smirnov test was used for evaluation of Gaussian distribution. Wilcoxon test was used to compare the means and standard deviations (SD) of PDFF in the two bone marrow and muscle compartments. Analysis for correlations of PDFF with age and BMI were done using Spearman´s rho. Furthermore, partial correlation testing was performed to adjust for BMI and age.

### Ethical approval

All procedures performed in this study using human participants were in accordance with the ethical standards with the institutional and the 1964 Helsinki declaration and its later amendments.

## Results

### Study population

Age was not significantly different between men and women (men: 36.9 ± 10.8 years, range: 23–61 years; women: 38.9 ± 14.7 years, range: 21–77 years; *p* = 0.980), neither was BMI (men: 26.4 ± 5.9 kg/m^2^, range: 19.9–44.5 kg/m^2^; women: 24.4 ± 5.6 kg/m^2^, range: 17.2–43.5 kg/m^2^; *p* = 0.057) (Table 1). 13 male and 33 female subjects were under 30 years, 14 male and 20 female subjects were between 31 and 49 years, and 4 male and 19 female subjects were older than 49 years.

**Table 1:** Subject characteristics (age and BMI) and PDFF values of muscular (gluteal and paraspinal muscles) and osseous compartments (sacrum and L5) are shown.

	subjects	n	Mean ± SD	p
age [years]	men	31	36.9 ± 10.8	0.980
	women	72	38.9 ± 14.7	
BMI [kg/m²]	men	31	26.4 ± 5.9	0.057
	women	72	24.4 ± 5.6	
PDFF_sacrum_ [%]	men	31	54.7 ± 12.0	0.903
	women	72	54.6 ± 12.1	
PDFF_gluteal muscles_ [%]	men	29	11.1 ± 10.7	0.167
	women	67	12.9 ± 9.8	
PDFF_L5_ [%]	men	31	38.8 ± 8.2	0.741
	women	72	40.7 ± 12.1	
PDFF_paraspinal muscles_ [%]	men	31	14.2 ± 7.8	**<0.0001**
	women	72	25.9 ± 9.3	

Parameters were compared between the two groups with Wilcoxon test (p-values). PDFF: proton density fat fraction, BMI: body mass index.

### PDFF measurements of the osseous and muscular compartments

**Figure 2:**
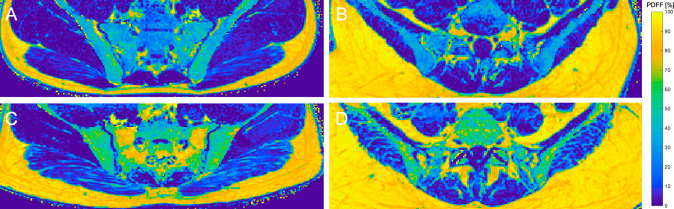
(**A**) Color-coded PDFF map of a 22-year-old male subject (BMI: 23.5 kg/m^2^, mean PDFF of sacrum: 37.3%; vertebral body L5: 22.2%; gluteal muscles: 5.8%; paraspinal: 10.7%). (**B**) Color-coded PDFF map of a 21-year-old female subject (BMI: 23.6 kg/m^2^, mean PDFF of sacrum: 36.8%; vertebral body L5: 28.0%; gluteal muscles: 6.3%; paraspinal: 13.9%). (**C**,**D**) show increased PDFF for the bone marrow compartments (sacrum and L5) as well as the local musculature compared to the younger subject in (**A**). (**C**) 45-year-old male subject (BMI: 26.8 m/kg^2^, mean PDFF of sacrum: 66.5%; gluteal muscles: 23.1%) (**D**) 57-year-old male subject (BMI: 24.0 kg/m^2^, vertebral body L5: 49.7%; paraspinal muscles: 10.9%). A color-coded scale bar displaying a spectrum from 0 to 100% can be seen at the right side. BMI: body mass index, PDFF: proton density fat fraction.

The average PDFF of the gluteal muscles was lower (*p* = 0.167) in men (11.1 ± 10.7%) than in women (12.9 ± 9.8%). PDFF of the paraspinal muscles showed significant differences between men (14.2 ± 7.8%) and women (25.9 ± 9.3%) (*p* < 0.0001). No significant differences between men and women could be detected in PDFF values of the sacrum (*p* = 0.903), vertebral body L5 (*p* = 0.741) and the gluteal muscles (*p* = 0.167) (Table 1). Representative color-coded PDFF maps of the osseous and muscular compartments of men and women are shown in Fig. 2.

### Correlations between sacrum, L5, gluteal and paraspinal musculature

**Figure 3:**
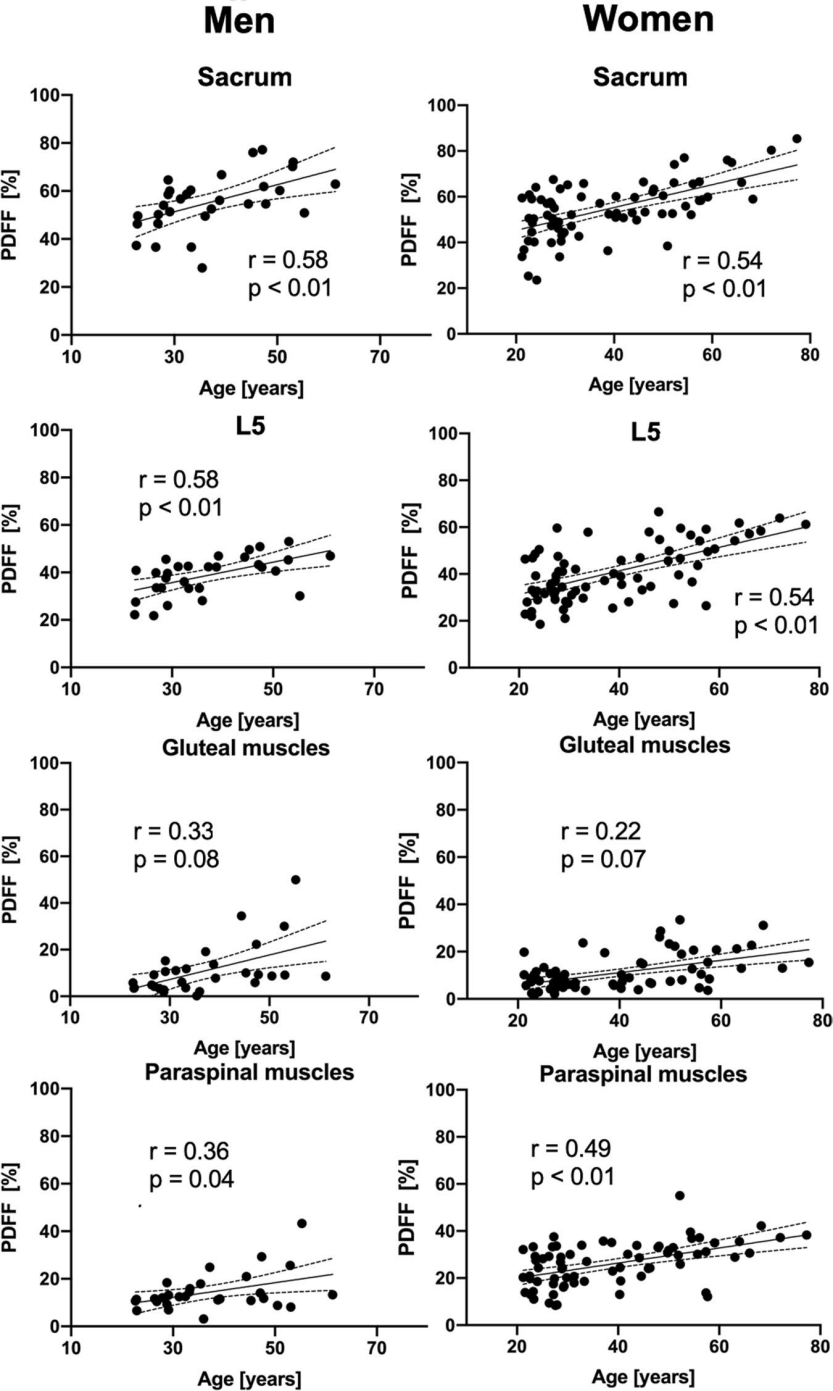
Scatter plots showing the PDFF of the sacrum, L5, the gluteal and paraspinal muscles against age for men and women, respectively. The areas between the dotted lines represent the 95% confidence band of the best-fit line. PDFF: proton density fat fraction. n.s.: not significant.

Correlation with age was significant for the PDFF measurements of the sacrum (men: *r* = 0.58, *p* < 0.01; women: *r* = 0.54, *p* < 0.01), L5 (men: *r* = 0.58, *p* < 0.01; women: *r* = 0.54, *p* < 0.01) and the paraspinal muscles (men: *r* = 0.36, *p* = 0.04; women: *r* = 0.49, *p* < 0.01) (Table 2). PDFF of the gluteal muscle showed no significant correlation with age (men: *r* = 0.33, *p* = 0.082; women: *r* = 0.22, *p* = 0.071). BMI correlated significantly with the PDFF measurements in paraspinal muscles (men: *r* = 0.456, *p* = 0.01; women: *r* = 0.334, *p* < 0.01) in both genders (Table 2 and Fig. 3).

**Table 2:** Correlation of subjects‘ characteristics (age and BMI) and PDFF values for muscular and osseous compartments in men and women.

		PDFF_sacrum_ [%]	PDFF_gluteal muscles_ [%]	PDFF_L5_ [%]	PDFF_paraspinal muscles_ [%]
Age [years]	male	0.58, <0.01	n.s.	0.58, <0.01	0.36, 0.04
	female	0.54, <0.01	n.s.	0.54, <0.01	0.49, <0.01
BMI [kg/m^2^]	male	n.s.	n.s	n.s.	0.46, 0.01
	female	n.s.	n.s.	0.24, 0.04	0.33, <0.01

Parameters were compared with Spearman’s rho test (Spearman’s rho, p values are given for each significant correlation). PDFF: proton density fat fraction, BMI: body mass index.

### Partial correlation testing

Partial correlation testing with age and BMI as control variables revealed significant correlations (*p* < 0.05) only between the two osseous compartments (men: *r* = 0.61, women: *r* = 0.75) in both genders, but not between the gluteal and paraspinal muscle compartments as well as the bone marrow and muscle compartments (*p* > 0.05).

## Discussion

In this analysis chemical shift encoding-based water-fat MRI was used to quantitatively assess PDFF of the sacral and lower lumbar vertebral bone marrow and the local musculature across a broad age- and BMI range in men and women. Significant positive correlations between age and PDFF in both osseous as well as the paraspinal muscle compartments could be detected, while the paraspinal muscle PDFF also correlated with BMI. Applying partial correlation testing with BMI and age as control variables, significant correlations for the bone marrow compartments were preserved in men and women.

Distinct intra- and inter-individual PDFF distribution patterns were detected in the present analysis, with higher PDFF values in the sacrum than in L5. This increase in bone marrow PDFF from cranial to caudal at the vertebral column is in line with results from previous studies on the lumbar vertebral bone marrow^22,31^. Furthermore, the present analysis found increasing bone marrow and paraspinal muscle PDFF values with advancing age, corroborating results from other studies showing that subtle tissue alterations in the analyzed body compartments occur in healthy subjects due to aging processes from childhood to senescence^32,33^. Visualizing the pathophysiologic processes occurring in healthy subjects due to aging and understanding the intra- and inter-individual variations of the regional degree of fatty infiltration allows a better differentiation between beginning disease manifestations and normal physiologic conditions. Moreover, a deeper knowledge of these processes on the lower lumbar and sacral level might give further insights regarding complications of osteoporosis such as sacral insufficiency fractures.

The results of the present analysis point to a distinct and differential composition pattern of bone marrow and local musculature at the upper pelvic/ gluteal region. Sollmann *et al*. reported significant correlations between PDFF of the lumbar bone marrow and the local musculature in postmenopausal, but not in premenopausal women^12^. This seemingly contradictory finding may be explained by the different anatomical locations investigated and the fact that our analysis comprised a relatively young study population as compared to the postmenopausal women included by Sollmann *et al*.

An inverse correlation between age and bone mineral density (BMD) was shown by several groups in the past^34,35^. In older adults, higher vertebral bone marrow fat has been shown to be associated with lower trabecular BMD and vertebral fractures^36^. Another study reported no detectable correlation of BMD and intramuscular fat content in the gluteal in contrast to thigh muscles in hip fracture patients^14^. The present analysis endorses the hypothesis that for gluteal and paraspinal muscle fat infiltration, different pathomechanisms seem to be involved than for changing bone marrow composition in the course of aging at the lower lumbar and sacral level. Identifying the precise underlying processes synchronizing the altering bone marrow and muscular tissue composition during a lifespan still remains a challenge surpassing the scope of imaging alone. The specific fat deposition patterns in muscle and bone marrow elucidated in this analysis are further supported by results of investigations on clinical aspects of local compositional alterations in these tissues in fracture patients, where distinct increased levels of intramuscular fat and decreased BMD could be detected^5,14^.

Finally, bone marrow fat content and fatty infiltration of the paraspinal musculature have been shown to correlate with subcutaneous and visceral fat depots, which are well known to boost the risk of metabolic disturbances^37–40^. In the present analysis, the paraspinal muscle PDFF correlated with BMI, which points in the same direction. Adding different physiological and ectopic fat depots to a multimodal and multiparametric assessment protocol enables the clinician to identify the obese phenotype, evaluate the individual risk of (obese) subjects more accurately and to initiate early interventions. Against the background of an increasing awareness of sarcopenic obesity leading to loss of muscle mass and functional impairment in the elderly, the identification of subjects at risk is of high clinical importance^13,14,41–43^, and deeper knowledge about the processes altering bone marrow and muscular tissue composition and their relationship might prove useful for this.

A possible field of clinical implementation might be adding the presented sequence with a comparably short scan time of two minutes to the scanning protocol of elderly patients at risk for obesity or sarcopenic obesity. Elevated levels of bone marrow and intramuscular PDFF might add quantitative information to the holistic and multi-parametric fracture risk or metabolic syndrome risk assessment. In the light of the potential clinical implementation in primary and secondary prevention the reported data might serve as reference standard for pathology delineation.

There are certain inherent strengths and methodological limitations regarding the design of the present analysis, which are elucidated in the following. First, the large cohort size provides a good basis for age- and BMI-dependent analysis of PDFF variations. However, the men to women ratio as well as the different age groups could be balanced more equally in future studies, as elderly female subjects and male subjects in general are underrepresented. Second, we performed this study with a cross-sectional design. Thus, no intraindividual dynamics over a certain time period can be evaluated. Longitudinal studies with at least one follow-up scan or several scans at defined time points should be concepted to further investigate the temporal dynamics of PDFF changes in individuals. Third, no BMD measurements were performed. However, the reciprocal connection of BMD and PDFF values has been shown by previous studies, amongst others by Kühn *et al*.^44^. Lastly, the analysis lacks information about potential confounding factors for bone marrow and muscle composition such as physical activity, nutrition or smoking habits. Future analyses should include these potential confounders.

In summary, this analysis showed that variations in sacral and lumbar bone marrow composition detected by using chemical shift encoding-based water-fat MRI were age-related, while only the paraspinal muscle fat content correlated with BMI in both genders. However, bone marrow and muscle fat were not significantly associated with each other at the sacral and lower lumbar spine level. In conclusion, this might point to different underlying pathophysiological fat infiltration patterns within the different compartments, but further clinical studies are needed to give this theory more evidence and exclude confounding aspects like smoking habits or level of physical activity.
